# LTSS Direct Care Worker Employment During COVID-19

**DOI:** 10.1093/geroni/igab046.604

**Published:** 2021-12-17

**Authors:** Janette Dill, Bianca Frogner

**Affiliations:** 1 University of Minnesota, Minneapolis, Minnesota, United States; 2 University of Washington, Seattle, Washington, United States

## Abstract

The crisis of COVID-19 in long-term care services and supports (LTSS) has brought attention to challenges in staffing long-term care organizations, as shortages of direct care workers led to a dramatic inability to provide needed care for many residents in nursing homes and other residential care settings. In this study, we examine unemployment among LTSS direct care workers during the crisis and recovery. This study uses monthly data from January 2019 to December 2020 from the Current Population Survey, a monthly household survey collected by the Bureau of Labor Statistics, and we compare an individual’s 2019 monthly employment patterns to their 2020 monthly employment. Long-term care workers had an unemployment rate of 2.8% in April 2020, when unemployment rates in the US reached a peak; however, new unemployment among long-term care workers has not declined as consistently as in other settings. Female health care workers were significantly more likely to be unemployed compared to their male counterparts, a trend that is consistent with the overall economy, and workers who earned the lowest wages were more likely to have transitioned to unemployment. COVID-19 has added significant complexity to the provision of direct care services, making LTSS a hazardous place to work. Concerns remain about unemployment in long-term care where demand for workers remains high; additional measures need to be taken to ensure that direct care workers have the resources they need to remain employed.

